# Erchen Decoction Mitigates Lipid Metabolism Disorder by the Regulation of PPAR*γ* and LPL Gene in a High-Fat Diet C57BL/6 Mice Model

**DOI:** 10.1155/2020/9102475

**Published:** 2020-03-10

**Authors:** Mengting Zhang, Yanfei Shao, Bizhen Gao, Jicheng Chen, Ping Zhang, Yujie Hu, Shanshan Ding

**Affiliations:** ^1^The First Affiliated Hospital of Xiamen University, Xiamen 361003, China; ^2^Fujian University of Traditional Chinese Medicine, Fuzhou 350122, China; ^3^Fujian Key Laboratory of TCM Health State, Fujian University of Traditional Chinese Medicine, Fuzhou 350122, China; ^4^Department of Endocrinology, Quanzhou Hospital of Traditional Chinese Medicine, Quanzhou 362002, China

## Abstract

Erchen decoction (ECD) is a common treatment prescribed in traditional Chinese medicine (TCM) clinics, which has remarkable efficacy in the treatment of obesity, fatty liver, hyperlipidemia, diabetes, and other diseases caused by phlegm. In this study, we investigated the effect that ECD had on the lipid metabolism induced by high-fat diet in C57BL/6 mice. Body weight, body length, and abdominal circumference were detected. Blood lipid content was measured via biochemical assay kit. The gene and protein expression of PPAR*γ* and LPL in visceral fat and skeletal muscle of mice was measured by real-time PCR and western blot. The research discovered that the phlegm-resolving effect that ECD had on high-fat diet mice was mainly manifested as reduced body weight, Lee's index, abdominal circumference, and level of TG and TC. Meanwhile, we observed significantly increased PPAR*γ* mRNA and protein level in visceral fat and PPAR*γ* and LPL protein level in skeletal muscle in the ECD group. Contrarily, a decrease in PPAR*γ* mRNA level in skeletal muscle in the ECD group was observed. Therefore, we speculate that ECD regulates the lipid metabolic disorder by decreasing the blood lipid level. Moreover, the potential molecular mechanism of ECD is to promote the expression of PPAR*γ* in visceral fat and skeletal muscle and the expression of LPL in skeletal muscle.

## 1. Introduction

Changes in diet habits in modern society has accelerated the development of diseases such as obesity, fatty liver, and hyperlipidemia, which threatens people's health. A number of studies have found [1, 2] that phlegm is the main pathological factor of obesity, hyperlipidemia, and other diseases in traditional Chinese medicine (TCM).

Erchen decoction (ECD), originated from Taiping Huimin Formula Bureau, is commonly prescribed in TCM clinics for its remarkable effect in the treatment of obesity, fatty liver, hyperlipidemia, diabetes, and other diseases caused by phlegm [3–6]. Its roles include enhancing dryness and dampness, removing phlegm, regulating qi, and moderating qi. In the prescription, *Pinellia ternata* is the monarch medicine, which can not only dry dampness and dissipate phlegm but also reduce adverse reactions with the stomach. Tangerine peel, which is compatible with regulating qi and stagnation, is the minster medicine. It can enhance the power of dryness and dampness and dissipate phlegm, embodying the idea of regulating qi before treating phlegm. *Poria cocos* is used to invigorate spleen and permeate dampness, while licorice is used as an adjunct to invigorate spleen and neutralize and harmonize various medicines. In animal experiments [7, 8], it also showed excellent phlegm-resolving effect. However, the mechanism of ECD in regulating lipid metabolism disorder is still unclear.

Peroxisome proliferator-activated receptor gamma (PPAR*γ*) is located in the p25 region of chromosome 3 and is a class of ligand-activated nuclear transcription factors that play a key role in lipid metabolism, fat cell formation, and various biological processes like inflammation. PPAR*γ* can be expressed in fat, kidney, spleen, and skeletal muscle, although most current studies concentrate on fat and skeletal muscle [9]. Lipoprotein lipase (LPL), located in the short arm 2 region 2 of chromosome 8, is a key enzyme in lipid metabolism and transportation. It is expressed in a variety of tissues, such as the heart, skeletal muscle, fat, lung, kidney, thymus, brain, and liver, with the highest content in skeletal muscle and visceral fat [10]. LPL and PPAR*γ* are both genes that promote lipid catabolism. Studies have found that PPAR*γ* can regulate the expression of lipoclastic differentiation related factors such as LPL and promote lipid oxidation, thereby regulating the fatty acid metabolism in fat and muscle tissues. Hence, this experiment investigated the effect that ECD had on lipid metabolism in mice with a high-fat diet. Moreover, the expression levels of mRNA and protein in PPAR*γ* and LPL were detected in the skeletal muscle tissue and visceral fat to explore the possible molecular mechanism that ECD plays in eliminating phlegm.

## 2. Materials and Methods

### 2.1. Experimental Animals

32 SPF adult male C57BL/6 mice, weighing 20 g ± 2 g, were purchased from Shanghai Slack Laboratory Animal Company, experimental animal license number: SCXK (Shanghai) 2012-0002. The mice were housed in an SPF barrier system of the Experimental Animal Center of Fujian University of TCM, under controlled temperature (24°C ± 2°C), humidity (55% ± 10%), and eating and drinking ad lib, with a daylight period of 08:00–20:00. The ordinary diet contained 5% fat, 23% protein, and 53% carbohydrate with total calorific value 25 kJ/kg (Shanghai Slac Laboratory Animal Company, Shanghai, China), whereas the high-fat diet contained 34.9% fat (60% of calories), 26.3% carbohydrates (20% of calories), and 26.2% protein (20% of calories) as well as fiber, vitamins, and minerals with total calorific value 21924 kJ/kg (D12492, Research Diets, New Brunswick, NJ, USA). All procedures in this study followed the “NIH Guide for the Care and Use of Laboratory Animals” (revised 2011).

### 2.2. Drugs and Preparations

The Erchen decoction was composed of *Pinellia ternata* 15 g, tangerine peel 15 g, *Poria cocos* 9 g, and *Glycyrrhiza uralensis* 4.5 g. All ingredients were purchased from the Guoyi hospital in Fujian University of TCM. Simvastatin dispersible tablets were used as a positive control (Xinke, 20 mg/tablet, Guangzhou Nanxin Pharmaceutical Co., Ltd., batch number: 3137506). According to the conventional dose conversion between mice and humans, the dose of ECD and simvastatin was 12 times the human standard dose, 8.7 g/(kg·d) and 2 mg/(kg·d). The ECD was prepared according to a conventional decoction method and concentrated to 50 mL by a rotary evaporation apparatus to obtain a crude drug content of 0.87 g/mL. The Western medicine was equipped with an aqueous solution of 10 mg of simvastatin dispersible tablet that was ground into a powder, and 50 mL of physiological saline was added. The concentration of the simvastatin aqueous solution was 0.2 mg/mL.

### 2.3. Reagents and Instruments

The reverse transcription kit (TAKARA, article number: RR037A), SYBR kit (TAKARA, article number: RR420A), LPL antibody (Abcam, article number: ab21356), PPAR*γ* antibody (Proteintech, article number: 16643-1-AP), internal reference GAPDH gene (Abcam, article number: ab70700), BIO-RAD Chemi Dox XRS + imaging system instrument, Eppendorf real-time PCR instrument (Mastercycler ep realplex4S), TECAN microplate reader, NBS ultra-low temperature refrigerator, SIGMA frozen high-speed centrifuge (3K30), Sanheng multipurpose electrophoresis instrument (DYY-12), and protein nucleic acid analyzer (DU650) were used.

### 2.4. Experimental Design

Modeling and interventions were performed 1 week after adaptive feeding. The mice were randomly divided into a control group (NFD, *n* = 8) and high-fat group (HFD, *n* = 32) via a random number table. They were subjected to either a normal feed or high-fat diet. After 10 weeks, the high-fat group was randomly divided into the model group (HFD), the ECD group (ECD), and the simvastatin group (SVN). The mice in the ECD group were given 8.7 g/(kg·d) of ECD and the Western medicine group was given simvastatin 2 mg/(kg·d). The NFD group and the HFD were intragastrically administered distilled water for 4 weeks. The gavage volume of each group was 10 ml/kg. During the experiment, the groups that were not in the NFD were provided with the high-fat diet. The body weight, body length, and abdominal circumference of the mice were recorded every two weeks. The formula of Lee's index is as follows: Lee's index = (final body mass × 1000)^1/3^/body length.

At 4^th^ week and 10^th^ week, the mice were fasted overnight and anesthetized, and the blood was collected from the orbital sinus of mice for serum TG and TC analysis. After 14 weeks, the mice were euthanized and the abdominal aorta blood samples were taken using a coagulation tube. The visceral fat and skeletal muscles were removed, rapidly frozen with liquid nitrogen, and transferred to a −80°C refrigerator to detect PPAR*γ*, LPL mRNA, and protein expression.

### 2.5. Detection of Lipid Content in Serum

The separation of serum and serum TG and TC were measured following the instructions of the biochemical assay kit (Nanjing Institute of Bioengineering).

### 2.6. Real-Time PCR Detection of PPAR*γ* and LPL mRNA Expression Levels

Total RNA from visceral fat (500–800 mg/sample) and skeletal muscle (50–100 mg/sample) was extracted via the Trizol method, where its purity and concentration were measured (Nanodrop 2000 micro-ultraviolet spectrophotometer). The amplification conditions were predenaturation at 95°C for 30 s, two cycles of reactions (95°C, 30 s; 60°C, 30 s) for 40 cycles, and the dissolution curve. The *β*-actin was used as the internal reference gene to obtain the Ct value of the target gene. The relative expression amount was calculated as follows: −△△ct = the mean value of △ct of the experimental group; the mean value of △ct of the control group was subtracted and then converted to 2^−△△ct^, which was the amount of gene expression in the experimental group relative to the control group. The primers were synthesized by Shanghai Jierui Company and the sequence is described in Table 1.

### 2.7. Western Blotting Detection of PPAR*γ* and LPL Protein Expression Levels

Visceral fat (200–300 mg/sample, each group chose six samples at random) and skeletal muscle (50–100 mg/sample, each group chose six samples at random) homogenate, protein extraction, and BCA assay protein concentration were used to calculate the loading volume. The SDS-PAGE gel was loaded on each well with 35 *μ*g of the protein sample, at 20 V for 10 min. The voltage used for the concentrated glue was at 70 V for 30 min. The voltage used to separate the glue was 100 V for 1 h. The voltage was at 20 V for 10 min and then transferred to the PVDF membrane and sealed with 5% milk for 1 h. The mice LPL monoclonal antibody is diluted with the ratio of 1 : 2000 diluted mice LPL monoclonal antibody (plus 1 : 2200 while the mouse PPAR*γ* polyclonal antibody is diluted with the ratio of 1 : 2200. Both of the two antibodies are shaken overnight on the membrane at the temperature of 4°C. And then, the membrane was washed three times for ten minutes each.. A solution of 1 : 5000 goat anti-mouse antibody was added to incubate the LPL while a 1 : 5000 goat anti-rabbit antibody solution was added to incubate the GAPDH. Both were shaken at 37°C for 1 h on a constant temperature shaker before being washed three times with TBST for 10 min. Supersensitive ECL chemiluminescence reagents were developed for imaging on a BIO-RAD Chemi Dox XRS^+^ imager. The gray values of the imaged image were read and analyzed by software.

### 2.8. Statistical Analysis

All data conformed to normal distribution and were expressed as $\bar{x}\;\pm \;s$. Two groups of measurement data were analyzed by SPSS21.0 software via a *t*-test. Multigroup measurement data were analyzed via one-way analysis of variance and multiple sets of rank sum tests. *P* < 0.05 was considered statistically significant.

## 3. Results

### 3.1. ECD Reduced the Body Weight, Lee's Index, and Abdominal Circumference in High-Fat Diet Mice

Between the 2^nd^ and the 10^th^ week, the body weight, Lee's index, and the abdominal circumference of the HFD group were significantly higher than those of the NFD group (*P* < 0.01). After the medication intervention, the body weight, Lee's index, and the abdominal circumference of the ECD group and the SVN group were significantly lower than those of the HFD group at week 12 and 14 (*P* < 0.05, *P* < 0.01) (Tables 2–5). These dates had been published [11, 12].

### 3.2. ECD Reduced the Level of TG and TC in High-Fat Diet Mice

On the 10^th^ week, TG and TC in the HFD group were significantly higher than those in the NFD group (*P* < 0.05). After the intervention, the TG content in the SVN group was similar to the NFD group at the end of the 14^th^ week (*P* > 0.05), whereas the other groups exhibited significantly higher content than the NFD group (*P* < 0.05). The TG and TC of ECD and SVN group significantly decreased (*P* < 0.01) compared to the HFD group (Tables 6 and 7). These dates had been published [11, 12].

### 3.3. ECD Increased the Expression of PPAR*γ* in Visceral Fat

**Figure 1:**
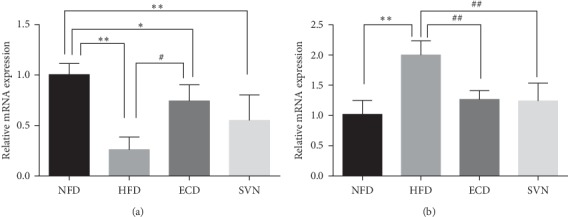
The mRNA expression of PPAR*γ*: (a) visceral adipose tissue; (b) skeletal muscle tissue. Compared with the NFD group, ^*∗*^*P* < 0.05, ^*∗∗*^*P* < 0.01; compared with the HFD group, ^#^*P* < 0.05, ^##^*P* < 0.01.

**Figure 2:**
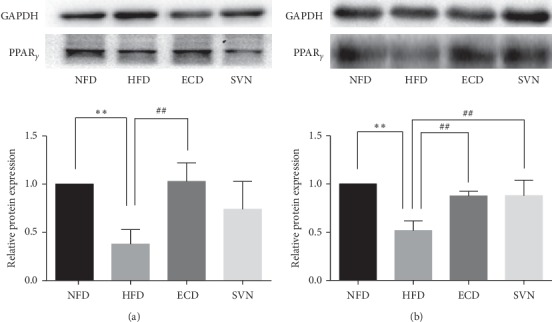
The protein expression of PPAR*γ*: (a) visceral adipose tissue; (b) skeletal muscle tissue. Compared with the NFD group, ^*∗∗*^*P* < 0.01; compared with the HFD group, ^#^*P* < 0.05, ^##^*P* < 0.01.

The expression of PPAR*γ* mRNA and protein in the HFD group was significantly lower than that in the NFD group (*P* < 0.01). The expression of PPAR*γ* mRNA and protein increased in the ECD group compared to the HFD group (*P* < 0.05, *P* < 0.01). The expressions of PPAR*γ* mRNA and protein in the SVN group were higher than those in the HFD group, but the difference was not statistically significant (*P* > 0.05) (Figures 1(a) and 2(a)).

### 3.4. ECD Increases the Expression of PPAR*γ* Protein and Reduced the Expression of PPAR*γ* mRNA in Skeletal Muscle

The expression of PPAR*γ* mRNA in the HFD group significantly increased (*P* < 0.01) but the expression of the protein significantly decreased (*P* < 0.01) compared with the NFD group. Compared with the HFD group, the expression of PPAR*γ* mRNA in the ECD group significantly decreased (*P* < 0.01) and the protein expression significantly increased (*P* < 0.05). Meanwhile, the expression of PPAR*γ* mRNA in the SVN group significantly decreased (*P* < 0.01) and the protein expression significantly increased (*P* < 0.05) (Figures 1(b) and 2(b)).

### 3.5. ECD Had No Effect on LPL Gene Expression in Visceral Fat

**Figure 3:**
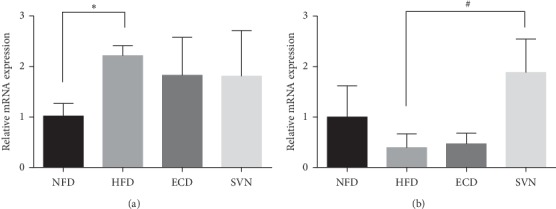
The mRNA expression of LPL: (a) visceral adipose tissue; (b) skeletal muscle tissue. Compared with the NFD group, ^*∗*^*P* < 0.05; compared with the HFD group, ^#^*P* < 0.05.

**Figure 4:**
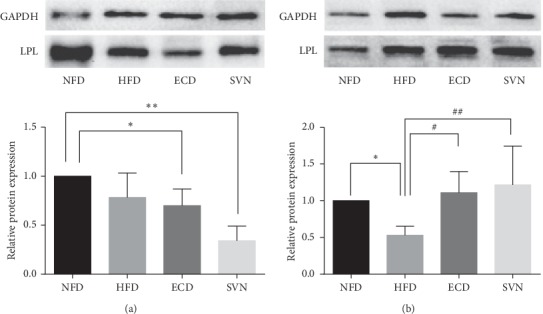
The protein expression of LPL: (a) visceral adipose tissue; (b) skeletal muscle tissue. Compared with the NFD group, ^*∗∗*^*P* < 0.05; compared with the HFD group, ^#^*P* < 0.05, ^##^*P* < 0.01.

The expression of LPL mRNA in the HFD group was higher than that in the NFD group (*P* < 0.05) and the protein expression decreased (*P* > 0.05). Meanwhile, the expression of LPL mRNA and protein in the ECD and SVN groups was lower than that in the HFD group, but the difference was not markedly significant (*P* > 0.05) (Figures 3(a) and 4(a)).

### 3.6. ECD Increased the Expression of LPL Protein in Skeletal Muscle

The expression of LPL mRNA and protein decreased to a greater extent in the HFD group compared to the NFD group, but only the expression of protein was markedly significant (*P* < 0.05). Consistently, the levels of mRNA and protein expression of the ECD group increased more compared to the HFD group, but only the protein expression was statistically significant (*P* < 0.05). The expression of LPL mRNA and protein in the SVN group was significantly higher than that in the HFD group (*P* < 0.05, *P* < 0.01) (Figures 3(b) and 4(b)).

## 4. Discussion

High-fat diets easily result in lipid metabolism disorders and even lead to cardiovascular diseases, metabolic syndrome, and fatty liver [13]. The theory of TCM states that excessive fat is liable to produce dampness and phlegm, while obstruction of phlegm turbidity affects the function of the spleen and stomach. If our bodies cannot raise the spleen qi and decrease the stomach qi, then our bodies are unable to absorb the essence of foodstuff and transport body fluid. If body fluid accumulates, it is liable to produce phlegm, which leads to obstruction of blood vessels and hinders the operation of qi, blood stasis, and other pathological factors which promote the formation of phlegm turbidity. Modern Chinese medicine practitioners have found that ECD plays a significant role in regulating lipid metabolism in animals. Ding Shanshan et al. [7, 8] found that ECD can regulate the expression of Cav-1 mRNA in rats with a high-fat diet and can lower blood sugar, regulate lipid metabolism, and reduce insulin resistance. Gao et al. [14] found that ECD can increase the expression of CDKAL1 mRNA and protein in liver, visceral, and subcutaneous fat of mice and promote insulin secretion in mice.

The effect that the ECD intervention had on high-fat diet mice was evident in the significant reductions in body weight, Lee's index, and abdominal circumference of the mice and significantly decreased levels of TG and TC in the serum (*P* < 0.05). ECD may regulate the lipid metabolism of mice by decreasing body weight, Lee's index, abdominal circumference, and TG and TC levels. In order to further explore the mechanism of ECD that affects PPAR*γ* and LPL, the expression levels of PPAR*γ*, LPL mRNA, and protein in visceral fat and skeletal muscle of mice were detected.

PPAR*γ* is widely distributed in fat, skeletal muscle, heart muscle, and liver tissues. It is a key transcription factor in fat metabolism as it mediates the differentiation and maturation of fat cells as well as participates in fatty acid and cholesterol metabolism [15]. Studies have shown that PPAR*γ* activation can reduce fatty acids transported to the liver and muscle and reduce fat synthesis, which inhibits lipid metabolism [16]. LPL is widely distributed in liver tissues, the heart, visceral fat, and skeletal muscle tissue. LPL is a glycoprotein composed of 448 amino acid residues. LPL is synthesized in the rough endoplasmic reticulum of parenchyma cells and stored in the secretory tube. Studies have shown that LPL activity is unevenly distributed in different tissues, resulting in different lipid metabolism rates throughout the tissues [17]. Bickerton et al. [18] found that fatty acids decomposed by LPL in visceral fat were absorbed faster than plasma nonesterified fatty acids, which accelerated the deposition of fat, suggesting that LPL plays a role in promoting fat absorption in skeletal muscle. Decomposed fatty acids are released into the plasma, so LPL does not accelerate fat deposition in skeletal muscle, indicating differences in the fatty acid uptake pathways between visceral fat and skeletal muscle. Studies have found that activation of PPAR*γ* selectively increases LPL quality, activity, and expression of its partner LMF1 in SF [19]. PPAR*γ* can induce the expression of LPL in adipocytes, thereby promoting the lipid metabolism and lowering blood lipid levels [20]. In other research, PPAR*γ* is closely related to the expression of LPL in muscle tissue and is involved in the fatty acid metabolism in muscle tissue [21]. PPAR*γ* can affect the fatty acid metabolism by regulating the expression of target gene LPL [22–24], which improves plasma HDL, LDL, and TG levels.

This study found that the expression of PPAR*γ* mRNA in visceral fat was downregulated in the HFD group, whose mice were provided a high-fat diet (*P* < 0.05). The PPAR*γ* mRNA expression in skeletal muscle was upregulated (*P* < 0.05). The expression of LPL mRNA in the visceral fat of mice in the HFD group was upregulated (*P* < 0.05), and the expression of LPL mRNA in the skeletal muscle was downregulated, although the difference was not statistically significant (*P* > 0.05). After ECD intervention, the expression of PPAR*γ* mRNA and protein in the visceral fat of mice was higher than that in the HFD group (*P* < 0.05), and the expression of LPL mRNA and protein was lower than that in the HFD group (*P* > 0.05). In mouse skeletal muscle, the expression of PPAR*γ* mRNA was lower than that in the HFD group. The protein expression of PPAR*γ* was higher than that in the HFD group (*P* < 0.05). The expressions of LPL mRNA and protein were higher than those in the HFD group, and the protein expression was statistically significant (*P* < 0.05). According to the above experimental results, the ECD plays different roles in visceral fat and skeletal muscle. More concretely, in visceral fat tissue, it could increase the activity of PPAR*γ* in the visceral fat, inhibit the action of LPL to accelerate fat deposition in visceral fat, and improve the IR in the adipose tissue. It also increased the PPAR*γ* and LPL activity in skeletal muscle to accelerate lipid metabolism. This was consistent with the results of previous studies. This study clarified that ECD can reduce the body weight, abdominal circumference, and TG and TC levels in mice induced by a high-fat diet by affecting PPAR*γ* and LPL.

The results showed that the LPL mRNA expression was upregulated in visceral fat in the HFD group, while protein expression was downregulated. PPAR*γ* mRNA expression was upregulated and protein expression was downregulated in the skeletal muscle. The relationship between mRNA and protein is not strictly linear, but comprises more intrinsic and complex dependencies. Different regulatory mechanisms (such as synthesis and degradation rates) that affect protein synthesis and synthesis of synthetic mRNA explain the difference in the quantity of the two molecules [25]. And we will continue to investigate the AMPK phosphorylation change to completely explain how ECD regulates the lipid metabolism in the future.

## 5. Conclusion

Given that ECD is famous for treating diseases caused by phlegm in clinics, we observed the decrease of the body weight, Lee's index, abdominal circumference, and TG and TC levels in C57BL/6 mice after ECD intervention. Furthermore, from the perspective of molecular biology, the upregulation of skeletal muscle and visceral fat expression in LPL and PPAR*γ* illustrates the effectiveness of ECD in eliminating phlegm.

## Figures and Tables

**Table 1:** Real-time PCR primer sequences.

Gene	Sequence	Length (bp)
LPL	Upstream: 5′-ATTGACTCCCTGCTGAATGAAG-3′	146
	Downstream: 5′-CTCTTGGCTCTGACCTTGTTG-3′	
*β*-actin	Upstream: 5′-GGGAAATCGTGCGTGAC-3′	176
	Downstream: 5′-AGGCTGGAAAAGAGCCT-3′	
PPAR*γ*	Upstream: 5′-GGAGCCTAAGTTTGAGTTTGCTGTG-3′	153
	Downstream: 5′-TGCAGCAGGTTGTCTTGGATG-3′	

**Table 2:** Comparison of body weight (g) between normal and high-fat mice between weeks 2–10 ($\bar{x}\;\pm \;s$).

Group	Week 2	Week 4	Week 6	Week 8	Week 10
NFD	21.62 ± 1.13	25.05 ± 1.26	26.68 ± 1.01	28.21 ± 1.17	29.41 ± 1.26
HFD	24.85 ± 1.35^*∗∗*^	29.53 ± 2.40^*∗∗*^	34.11 ± 3.04^*∗∗*^	38.39 ± 3.98^*∗∗*^	42.00 ± 4.61^*∗∗*^

Compared with the NFD group, ^*∗∗*^*P* < 0.01.

**Table 3:** Comparison of Lee's index between normal and high-fat mice between weeks 2–10 ($\bar{x}\;\pm \;s$).

Group	Week 2	Week 4	Week 6	Week 8	Week 10

NFD	3.38 ± 0.05	3.30 ± 0.02	3.50 ± 0.05	3.48 ± 0.15	3.42 ± 0.14
HFD	3.33 ± 0.01	3.39 ± 0.01^*∗∗*^	3.52 ± 0.09^*∗∗*^	3.66 ± 0.12^*∗∗*^	3.77 ± 0.13^*∗∗*^

Compared with the NFD group, ^*∗∗*^*P* < 0.01.

**Table 4:** Comparison of abdominal circumference (cm) between normal and high-fat mice between weeks 2–10 ($\bar{x}\;\pm \;s$).

Group	Week 2	Week 4	Week 6	Week 8	Week 10

NFD	6.78 ± 0.33	6.97 ± 0.34	7.40 ± 0.21	7.80 ± 0.34	7.77 ± 0.34
HFD	7.20 ± 0.33^*∗∗*^	8.09 ± 0.37^*∗∗*^	8.60 ± 0.41^*∗∗*^	9.30 ± 0.39^*∗∗*^	9.41 ± 0.60^*∗∗*^

Compared with the NFD group, ^*∗∗*^*P* < 0.01.

**Table 5:** Comparison of body weight, Lee's index and abdominal circumference of each group between week 12 and week 14 ($\bar{x}\;\pm \;s$).

Group	Body weight (g)		Lee's index		Abdominal circumference (cm)	
	Week 12	Week 14	Week 12	Week 14	Week 12	Week 14

NFD	28.25 ± 1.15	28.94 ± 1.07	3.42 ± 0.06	3.41 ± 0.04	7.46 ± 0.30	7.77 ± 0.17
HFD	46.00 ± 3.09^*∗∗*^	48.31 ± 3.13^*∗∗*^	3.78 ± 0.09^*∗∗*^	3.83 ± 0.09^*∗∗*^	9.49 ± 0.33^*∗∗*^	10.13 ± 0.47^*∗∗*^
ECD	40.51 ± 3.15^*∗∗##*^	43.10 ± 3.38^*∗∗##*^	3.67 ± 0.05^*∗∗##*^	3.66 ± 0.16^*∗∗##*^	8.94 ± 0.34^*∗∗##*^	9.46 ± 0.35^*∗∗##*^
SVN	40.91 ± 2.86^*∗∗##*^	42.93 ± 3.16^*∗∗##*^	3.71 ± 0.06^*∗∗#*^	3.72 ± 0.07^*∗∗##*^	9.04 ± 0.33^*∗∗##*^	9.28 ± 0.40^*∗∗##*^

Compared with the NFD group, ^*∗∗*^*P* < 0.01; compared with the HFD group, ^#^*P* < 0.05, ^##^*P* < 0.01.

**Table 6:** Serum TG and TC in normal and high-fat groups between week 4 and week 10 (mmol·L^−1^).

Group	TG		TC	
	Week 4	Week 10	Week 4	Week 10

NFD	0.75 ± 0.19	0.85 ± 0.15	2.22 ± 0.14	2.31 ± 0.50
HFD	1.24 ± 0.30	1.54 ± 0.58^*∗*^	2.36 ± 0.29	3.14 ± 0.55^*∗*^

Compared with the NFD group, ^*∗*^*P* < 0.05.

**Table 7:** Comparison of serum TG and TC levels in each group on week 14 $\bar{x}\;\pm \;s$ (mmol·L^−1^).

Group	TG	TC
NFD	0.91 ± 0.10	2.64 ± 0.27
HFD	1.64 ± 0.30^*∗∗*^	4.66 ± 0.47^*∗∗*^
ECD	1.23 ± 0.09^*∗∗##*^	3.75 ± 0.45^*∗∗##*^
SVN	1.06 ± 0.06^##^	3.70 ± 0.49^*∗∗##*^

Compared with the NFD group, ^*∗*^*P* < 0.05, ^*∗∗*^*P* < 0.01; compared with the HFD, ^#^*P* < 0.05, ^##^*P* < 0.01.

## Data Availability

The data used to support the findings of this study are included within the article.
